# Evaluation of prognostic factors for late recurrence in clear cell renal carcinoma: an institutional study

**DOI:** 10.3389/fonc.2024.1446953

**Published:** 2024-10-07

**Authors:** Diana Voskuil-Galoş, Tudor Călinici, Andra Piciu, Adina Nemeş

**Affiliations:** ^1^ Department of Medical Oncology, The Oncology Institute Prof. Dr. Ion Chiricuţă, Cluj-Napoca, Romania; ^2^ Department of Medical Informatics and Biostatistics, University of Medicine and Pharmacy "Iuliu Haţieganu", Cluj-Napoca, Romania; ^3^ Department of Medical Oncology, University of Medicine and Pharmacy “Iuliu Haţieganu”, Cluj-Napoca, Romania

**Keywords:** recurrence, surgery, neutrophil to lymphocyte ratio, platelet to lymphocyte ratio, clear cell renal carcinoma (ccRCC)

## Abstract

**Background and objectives:**

Following nephrectomy with curative intent, a subset of patients diagnosed with non-metastatic renal cell carcinoma (nmRCC) will present late recurrences, with metastatic relapses after 5 years from the surgical intervention. The aim of this study is to evaluate the prevalence of late recurrences in Romanian patients with nmRCC that have undergone surgery and to assess the clinicopathological characteristics prognostic for late-relapse RCC.

**Materials and methods:**

This is a single-center, retrospective and observational study that analyzed patients with nmRCC with clear cell histology who underwent surgical resection of the primary tumor with curative intent. The patients included in the study were treated and further surveilled according to a personalized follow-up plan between January 2011 and December 2012 in The Oncology Institute "Prof. Dr. Ion Chiricuţă", Cluj-Napoca, Romania. Study endpoints included median disease-free survival (DFS), median overall survival (OS), as well as evaluation of possible prognostic factors indicative of late relapse.

**Results:**

In the study cohort (n=51), the median DFS was 46 months and median OS was 130 months. DFS was significantly correlated with the International Metastatic Renal Cell Carcinoma Database Consortium (IMDC) score (p=0.04, HR=2.48; 95% CI [1.02, 6.01]), neutrophil to lymphocyte ratio (NLR) (a higher NLR value was associated with a poorer DFS, p=0.035), tumor size (T4 tumors vs. T1 p<0.05, HR=9,81; 95% CI [2.65, 36.27]) and Fuhrman nuclear grade (Fuhrman grade 1 vs. Fuhrman grade 3 p<0.05, HR=4,16; 95% CI = [1.13,15.22]). Fifty one percent of the patients included experienced disease relapse. From this subgroup, a significant percentage of 42% patients presented disease recurrence after 60 months from nephrectomy. OS was correlated to IMDC score (p=0.049, HR=2.36; 95% CI [1, 5.58]) and Fuhrman nuclear grade (Fuhrman grade 1 vs. Fuhrman grade 3 p<0.05, HR=3,97; 95% CI [1.08, 14.54]).

**Conclusions:**

The results of this study support the previously presented biological behavior of RCC, demonstrating that late recurrences in RCC are not uncommon occurrences and patients with localized RCC should be followed up for a longer interval after the surgery for the primary tumor. In addition, the study strengthens the data supporting certain biomarkers as valuable prognostic factors determining survival outcomes of patients with RCC.

## 1 Introduction

Renal cell carcinoma (RCC) is responsible for over 4% of all new cancer cases diagnosed every year. With a median age at diagnosis of approximately 65 years, RCC affects twice as many men as women (1). Up to 70% of patients diagnosed with RCC have clear cell histology and may benefit from surgical or ablative interventions with curative intent when diagnosed in early stages (2). However, one third of all cases present distant metastasis at diagnosis and a subset of patients will develop metastasis after primary treatment for early-stage disease (3).

The pathological staging represents a key prognostic determinant, as patients diagnosed in early stages (I and II) experience a five-year survival rate of up to 90% (4). Indicators of a poor prognosis are low functional status score, low hemoglobin (Hb) levels, high neutrophil and platelet count, high values of serum lactate dehydrogenase, high levels of serum corrected calcium and personal history of diabetes mellitus (4, 5). Recent data suggests that the evaluation of neutrophil to lymphocyte ratio (NLR) and platelet to lymphocyte ratio (PLR) before tumor excision could select patients at risk of recurrence, with elevated NLR and PLR values being associated with a poor prognosis (6–8).

According to the clinical guidelines for RCC, following diagnosis of a suspicious kidney mass, proper clinical staging is mandatory. Stage I disease requires removal of mass through surgery (partial or radical nephrectomy) or ablative techniques (9). However, carefully selected patients could be considered for active surveillance (10). Patients diagnosed with stage II and III disease should undergo partial or radical nephrectomy (11), followed by surveillance or adjuvant treatment (12). Adjuvant treatment may be offered to patients with stage III high-risk disease or stage II disease and grade 4 tumor masses with clear cell histology with or without sarcomatoid characteristics (13). After primary treatment, patients with histologically confirmed clear cell RCC and early-stage disease verified through extensive imaging must follow an individualized follow-up protocol with a duration of 5 years or longer when clinically indicated.

RCC presents a particular biological characteristic revealed through late relapses occurring after a disease-free interval of more than 5 years (14). Studies have identified this behavior in a quarter of RCC patients who underwent nephrectomy as primary treatment (15). Further research has attempted to define predictive factors for late recurrences and describe the outcome of such cases, with somewhat conflicting results (16).

To the best of our knowledge, this is the first study on late recurrences of RCC conducted in the Romanian population. Our study aims to strenghten the data already available on the subject and extend the knowledge in order to better identify, surveil and treat patients at risk of developing late relapses. In addition, this study will evaluate potential predictive factors for relapse such as baseline Hb levels, NLR, PLR, tumor characteristics (T stage, Fuhrman nuclear grade) and the presence of comorbidities, notably diabetes mellitus.

## 2 Materials and methods

### 2.1 Study design

Our team conducted a non-interventional, retrospective, single-center study evaluating patients with non-metastatic renal cell carcinoma (nmRCC) with clear cell histology who were treated and/or surveilled according to a personalized follow-up protocol. The treatment and/or follow-up occurred between January 2011 and December 2012 at The Oncology Institute "Prof. Dr. Ion Chiricuţă" in Cluj-Napoca, Romania. The present research was verified in compliance with the principles of the Declaration of Helsinki, with all the participants providing written, informed consent. The study design was evaluated and approved by the Ethics Committee of The Oncology Institute "Prof. Dr. Ion Chiricuţă", Cluj-Napoca.

### 2.2 Inclusion and exclusion criteria

As inclusion criteria, patients included in the study required to be of an age above 18 years, with a histologically confirmed diagnosis of clear cell RCC without metastatic spread confirmed through imaging or histological studies at diagnosis. The investigation demanded that patients be biologically evaluated at diagnosis based on a predefined set of blood tests before undergoing surgery as primary treatment with curative intent. In addition, the research warranted patients to be included in a personalized follow-up program. Patients with ages below 18 years were not included in the study. Moreover, other histological subtypes of kidney cancer, as well as metastatic cases, were excluded from the investigation. Patients without baseline imaging or histological studies confirming the localized or locally-advanced stage were not further evaluated for study inclusion. Patients who did not undergo the biological evaluation of baseline Hb value, neutrophil count, platelet count, lymphocyte count and corrected calcium levels were excluded from additional patient record analysis. The cases that did not adhere to the follow-up program recommended by the attending physician were also excluded from the study. Data inconsistencies regarding follow-up visits (periodicity, history and physical examination, laboratory examinations, imaging studies) led to patient exclusion from the study data base and further statistical case analysis.

### 2.3 Patients

The research was designed to include patients with a minimum age of 18 years old at diagnosis, with a histological confirmation of RCC with clear cell histology and non-metastatic disease confirmed through imaging studies at diagnosis. Patients were required to have undergone surgical excision of the primary tumor with curative intent and further comply to individualized follow-up and surveillance.

Patients´ medical records were evaluated in order to collect baseline information: demographic data, Karnofsky performance status, comorbidities (diabetes mellitus in particular), blood tests analysis, complete histologic result, adjuvant treatment (when administered), date of progression, site of progression and date of decease (if applied).

Potential study participants defined based on the inclusion and exclusion criteria, presenting incomplete medical records or records displaying data inconsistencies were excluded from further evaluation, therefore justifying the limited sample size of 51 participants.

### 2.4 Treatment

As primary oncological treatment, patients underwent surgical intervention for removal of renal tumor mass with curative intent. A subset of carefully selected patients benefited from adjuvant treatment following radical nephrectomy, as per local protocol available during the time of treatment. After surgery, patients were subjected to a personalized follow-up.

### 2.5 Outcomes

Study endpoints included median disease-free survival (DFS) and median overall survival (OS). DFS was defined as the time interval elapsed between the surgical resection of the primary tumor with curative intent and first recorded disease progression or death of any cause. OS was defined as the time period between the date of diagnosis and the date of decease. In addition, the research aimed to define potential prognostic factors indicative of late recurrences: baseline Hb, NLR, PLR, tumoral features (T stage, Fuhrman grade), the presence of diabetes mellitus as associated comorbidity.

### 2.6 Statistical analysis

The data available was collected in an Excel worksheet. Survival data were analyzed using Rx64 v4.0.0. The patients alive at the time of analysis (16th February 2023) were censored. The survival curves were presented using the Kaplan-Meier method, and survival distributions were compared with the Log-rank test. The effects of the main clinical and pathological variables on OS and DFS were investigated with the Cox regression model.

## 3 Results

Our study evaluated data collected from 51 patients diagnosed with nmRCC with clear cell histology who underwent surgical resection of the kidney mass with curative intent. Patient and disease characteristics are summarized in **Table 1**. The study included 36 male and 15 female patients, with a median age at diagnosis of 55 years (range 34-76 years). The majority of patients, representing 69% of the patients included in this study, were diagnosed before the age of 60. Coexisting diabetes mellitus was evaluated in the patients included, with 7 patients presenting the condition. Regarding tumor localization, 47% of patients presented a left kidney mass, while 53% presented tumor in the right kidney. Tumor characteristics were further assessed: most patients presented with T1 stage tumors (39%), followed by T3 stage tumors (27.4%), with 25.5% and 7.9% of the remaining individuals featuring T2 and T4 stage tumors, respectively. Lymph node status could not be assessed in 33.4% of the cases, while 62.8% of the evaluated surgical specimens showed no nodal involvement. Microscopical lymphatic invasion was described in a minority of 4% of cases, while microscopical vascular invasion was confirmed in 9 of the cases representing 17.7%. Fuhrman grading system was used to categorize nuclear characteristics of tumor specimens as follows: the majority of patients evaluated presented grade 2 and 3 tumors (52.9% and 23.6%, respectively), with the rest of 19.6% displaying grade 1 and 3.9% displaying 4 features. Patients´ risk group was assessed by implementing the International Metastatic Renal Cell Carcinoma Database Consortium (IMDC) score, consisting of two clinical elements: less than 1 year between the time of diagnosis and systemic therapy and a Karnofsky performance status under 80% and four biological criteria as follows: Hb value below the lower limit of the normal range, neutrophil and platelet count exceeding the upper limit of the normal range and an elevated value of the corrected calcium (17). Patients with no prognostic factors were considered as being in a favorable risk-group, patients with one or two prognostic factors were considered as being in an intermediate risk-group and patients with three or more prognostic factors in an poor-risk group. The results showed that most patients were evaluated as favorable and intermediate risk group (66.6% and 31.4% respectively), with only one patient presenting poor prognostic features based on the IMDC score. After undergoing risk stratification and in accordance with local protocols to date (years 2011-2012), 4 patients were found eligible for adjuvant treatment after surgical removal of tumor mass. After surgical treatment, patients were subjected to personalized follow-up protocol as per local conduct. Twenty-six of the patients included in the study experienced disease progression, with 42.3% of them facing recurrence after 60 months of initial treatment. Of them, 7 patients were confirmed with locoregional recurrence in addition to distance disease spread. Regarding organ metastasis, the lung and bones were the most common sites of metastasis identified. A significant percentage of patients had metastasis involving lymph nodes, adrenal gland, contralateral kidney, brain and liver. A number of patients had metastatic growth in uncommon sites, such as the pancreas, the thyroid gland, or soft tissue. Four of the patients followed during the study developed metastasis in multiple organs (>3 organs).

**Table 1:** Patients’ characteristics.

Variable	Number of patients (n=51 patients)
Age	
Median (range)	55 (34-76)
<60	35 (68.6 %)
>=60	16 (31.4 %)
Sex	
M	36 (70.5 %)
F	15 (29.5 %)
Comorbidities – diabetes mellitus	
Yes	7 (13.7 %)
No	44 (86.3 %)
Tumor localization	
Left kidney	24 (47 %)
Right kidney	27 (53 %)
Tumor characteristics – T (tumor)	
1	20 (39.2 %)
2	13 (25.5 %)
3	14 (27.4 %)
4	4 (7.9 %)
Tumor characteristics – N (lymph nodes)	
X	17 (33.4 %)
0	32 (62.8 %)
1	2 (3.8 %)
Tumor characteristics – L (lymphatic invasion)	
0	49 (96 %)
1	2 (4 %)
Tumor characteristics – V (vascular invasion)	
0	42 (82.3 %)
1	9 (17.7 %)
Tumor characteristics – Fuhrman grade	
1	10 (19.6 %)
2	27 (52.9 %)
3	12 (23.6 %)
4	2 (3.9 %)
International Metastatic RCC Database Consortium risk group	
Favorable	34 (66.6 %)
Intermediate	16 (31.4 %)
Poor	1 (2 %)
Administration of adjuvant treatment	
Yes	4 (7.8 %)
No	47 (92.2 %)
Patients experience recurrence	
Yes	26 (51 %)
No	25 (49 %)
Disease free survival for patients with recurrence	Number of patients (n=26 patients)
<60 months	15 (57.7 %)
>= 60 months	11 (42.3 %)
Locoregional recurrence	Number of patients (n=26 patients)
Yes	7 (27 %)
No	19 (73 %)
Distant recurrence	Number of patients (n=26 patients)
Yes	26 (100 %)
No	0 (0 %)
Site of metastasis	Number of patients (n=26 patients)
Lung	19
Bone	10
Lymph nodes	4
Suprarenal gland	3
Contralateral kidney	3
Other (brain, soft tissue, pleura, pericardium, liver, pancreas, thyroid)	8
Multiple sites of metastasis (>3)	4

**Figure 1:**
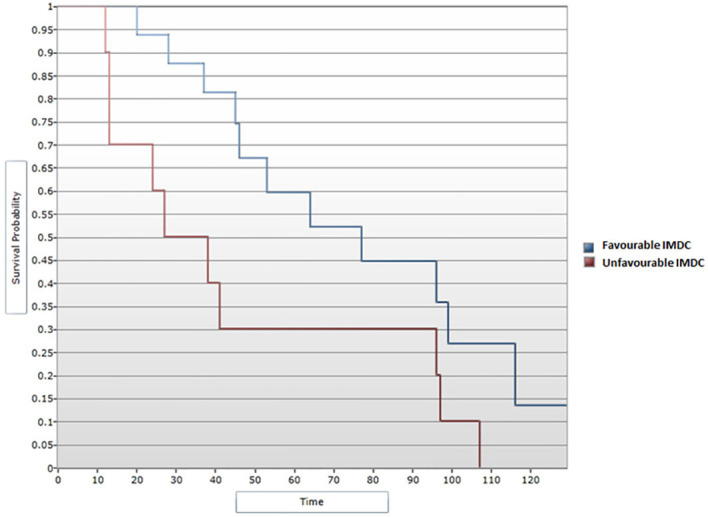
Survival analysis for IMDC score and PFS (Log-rank test).

**Figure 2:**
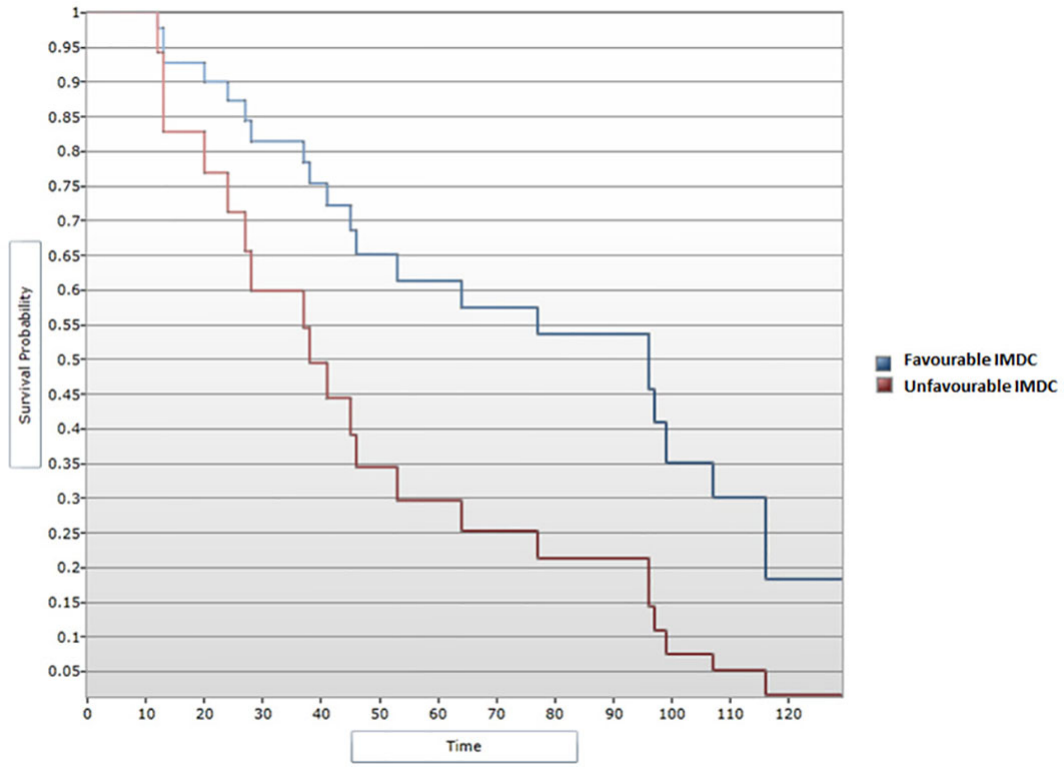
Cox regression analysis between IMDC score and DFS.

All data obtained from the medical records of patients included in this research were further included in the statistical analysis. A median DFS of 46 months was determined for the 26 patients included in the study who experienced disease progression following primary surgical treatment. Further, a favorable IMDC score was associated with an improved DFS in the univariate analysis, p=0.034 (**Figure 1**). In this analysis, given the small cohort, patients with an IMDC score evaluated as intermediate or poor were noted as presenting unfavorable IMDC score. A median DFS of 128 months was noted for patients with a favorable IMDC score vs. 97 months for those with unfavorable criteria. During the Cox regression analysis, the favorable IMDC score remained significantly correlated with an improved DFS, p=0.04 (HR=2.48, 95% CI [1.02, 6.01]) (**Figure 2**).

**Figure 3:**
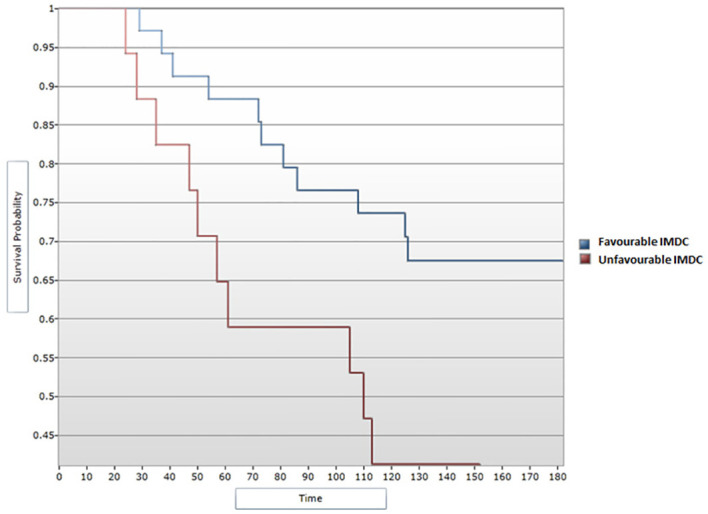
Survival analysis for IMDC score and OS (Log-rank test).

**Figure 4:**
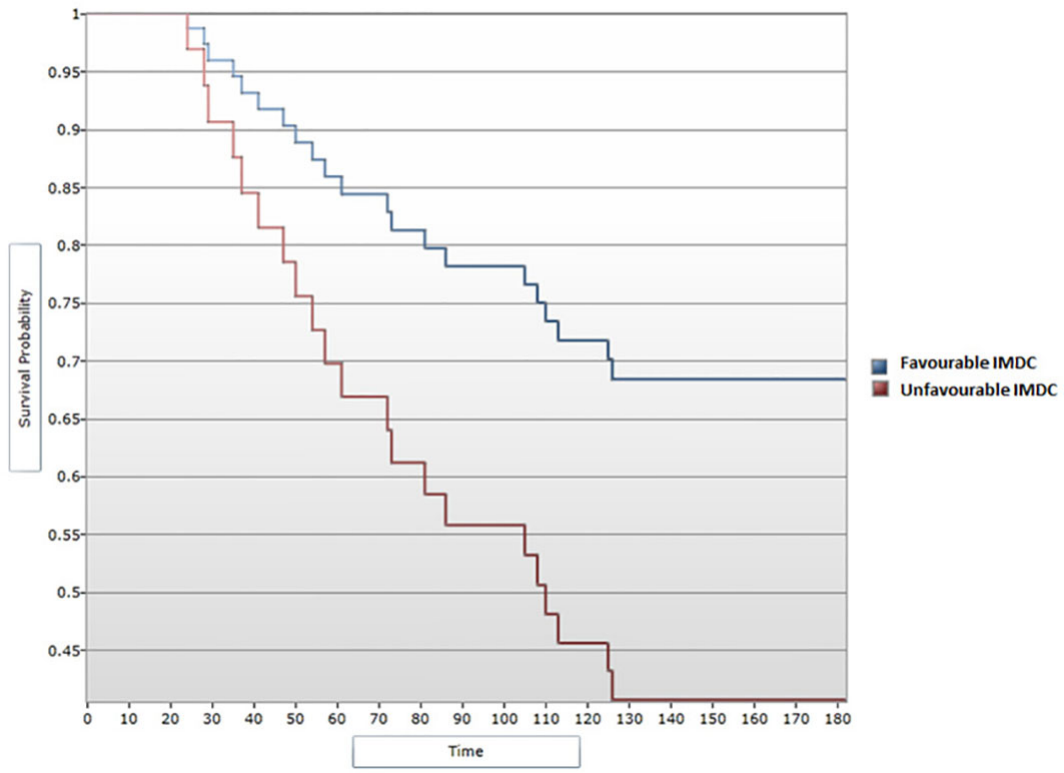
Cox regression analysis between IMDC score and OS.

A median OS of 130 months was calculated for all 51 patients included in the study. In the univariate analysis, a favorable IMDC score was significantly associated with an improved OS (p=0.042), with a median OS of 132 months for patients with a favorable IMDC score vs. 110 for those in the unfavorable score subgroup (**Figure 3**). The correlation remained statistically significant when applying the Cox regression model, with p=0.049 (HR=2.36, 95% CI [1, 5.58]) (**Figure 4**).

**Figure 5:**
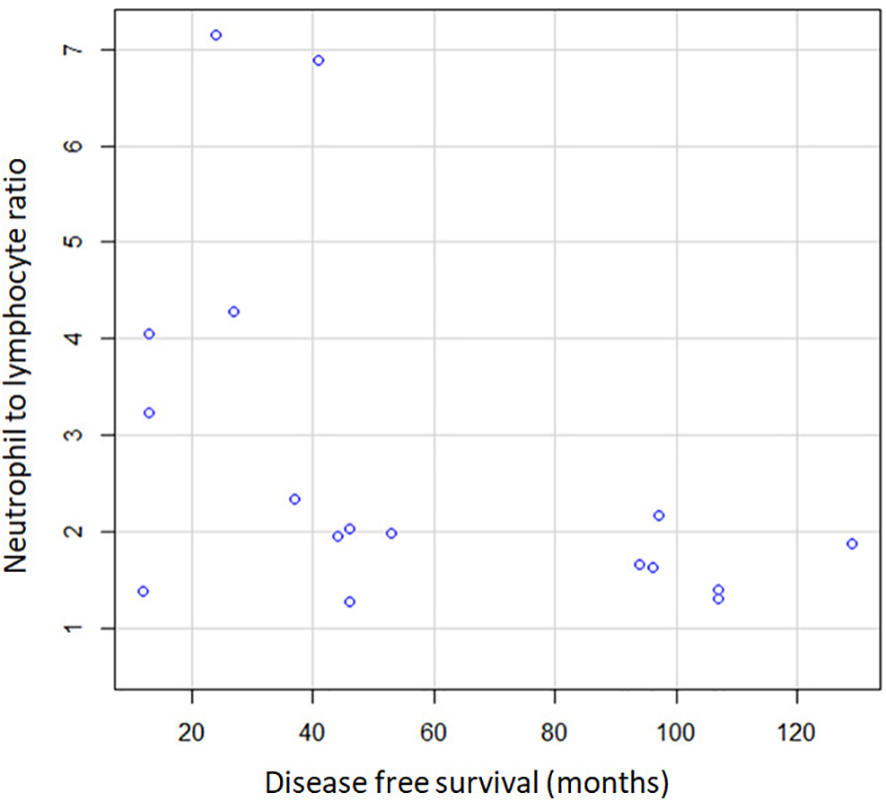
Correlation between NLR and DFS.

Additional potential predictors for OS and DFS were further evaluated, notably NLR, PLR and baseline Hb. Our analytical model described a linear regression in DFS parallel to NLR increase. Therefore, a higher NLR value was significantly associated with a poorer DFS (p=0.035) (**Figure 5**). In patients experiencing DFS up to 60 months since surgery with curative intent, the average NLR showed a value of 3.03. Contrastingly, in the subgroup of patients with a DFS longer than 60 months, the average NLR was 1.95. However, the correlation did not prove significant in terms of OS, with a p value higher than 0.05.

No statistically significant difference was noted in median DFS and OS when evaluating the correlation between PLR and survival outcomes (p>0.05). However, the average PLR in patients presenting with disease recurrence in the first 60 months since the surgical resection of the tumor mass was 138.83. This value proved to be notably higher that the PLR value of 125.65 corresponding to the subgroup of patients experiencing relapse after the 60 months mark.

Similarly, no statistically significant correlation could be identified between the Hb levels and DFS and OS (p>0.05). Nonetheless, a difference in the average value of Hb was noted between patients with DFS under 60 months and those with DFS exceeding 60 months. The analysis of the first subgroup reported an average Hb of 11.4 for female patients and 13.3 for male patients, compared to an average Hb value of 13.8 and 14, respectively, corresponding to the second group.

**Figure 6:**
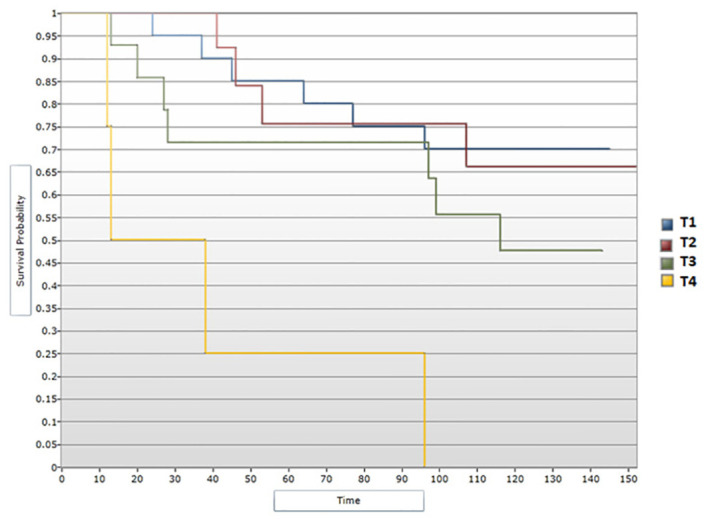
Survival analysis for T stage and DFS (Log-rank test).

**Figure 7:**
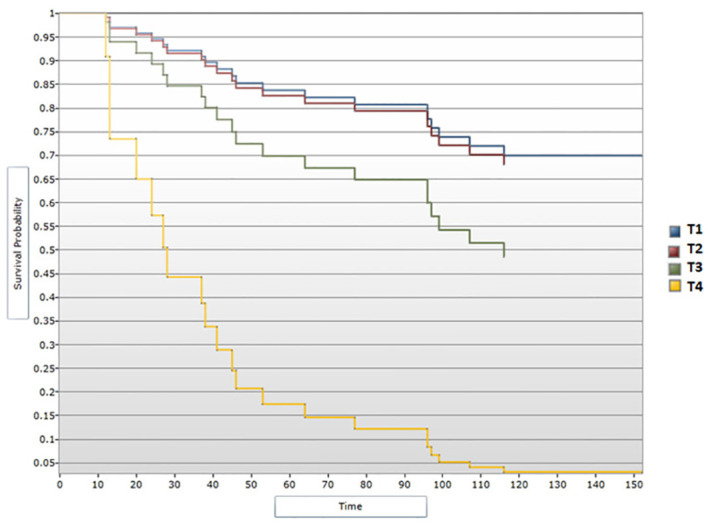
Cox regression analysis between T stage and DFS.

Tumor characteristics were additionally analyzed in order to outline survival profiles of patients included in the study. Median DFS showed a decline inversely proportional to tumor dimensions, correlation that proved to be significant statistically during the univariate analysis (p<0.05) (**Figure 6**). Patients with T1 tumors presented a median DFS of 131 months, those with T2 tumors had a median DFS of 125 months and patients with T3 disease had a median DFS of 107.5 months. Of note, patients presenting with T4 tumors showed a decreased median DFS of 25.5 months. Cox regression model confirmed the notable difference in DFS between T4 tumors vs. T1 (HR=9,81; 95% CI [2.65, 36.27] p<0.05) (**Figure 7**). However, tumor dimensions did not prove to impact OS outcomes. OS analysis based on tumor stage subgroups demonstrated minimal differences between median OS of patients with T1 tumors vs. T2 and T3 tumors (132 months, 130 months and 127 months respectively), with the T4 disease subgroup experiencing an interesting median OS of 59 months.

**Figure 8:**
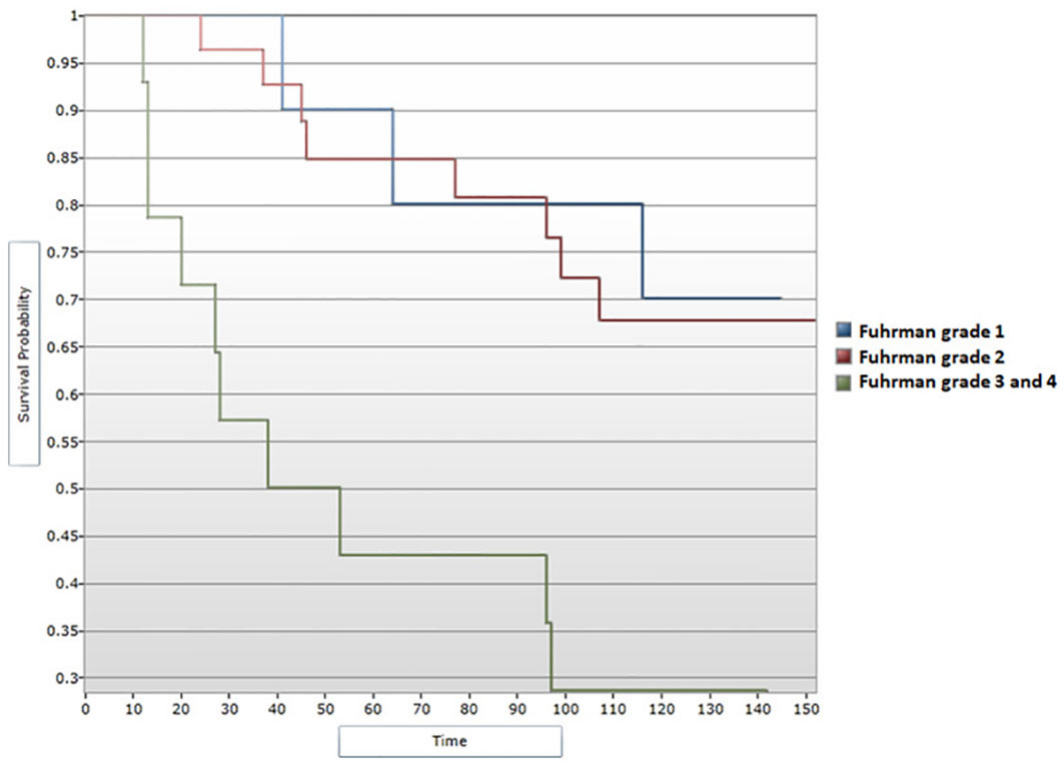
Survival analysis for Fuhrman grade and DFS (Log-rank test).

**Figure 9:**
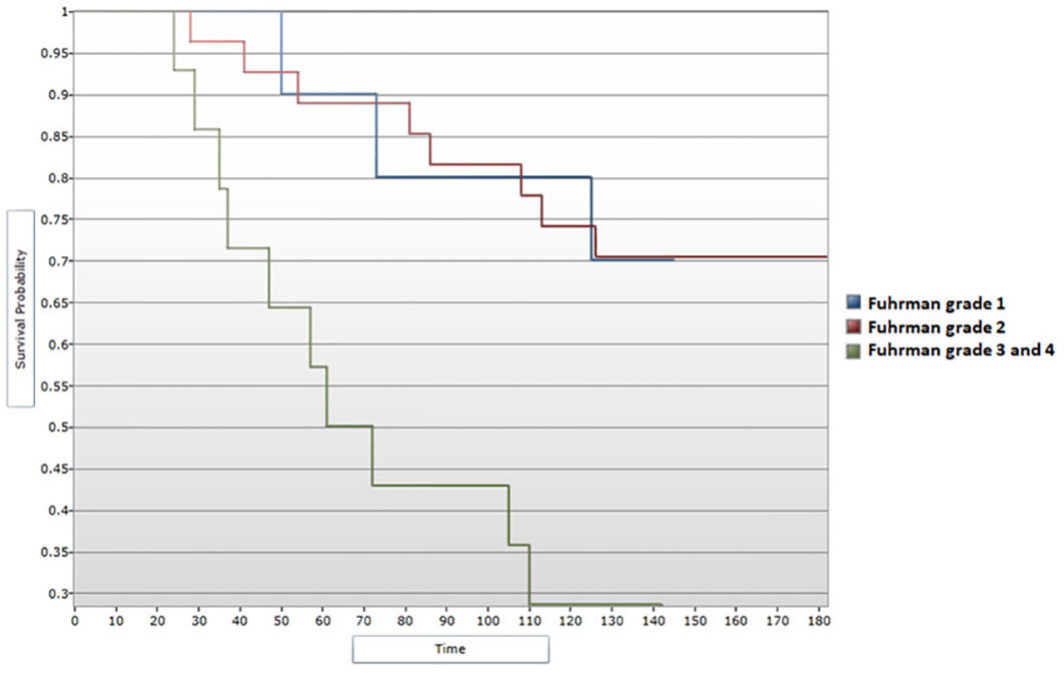
Survival analysis for Fuhrman grade and OS (Log-rank test).

**Figure 10:**
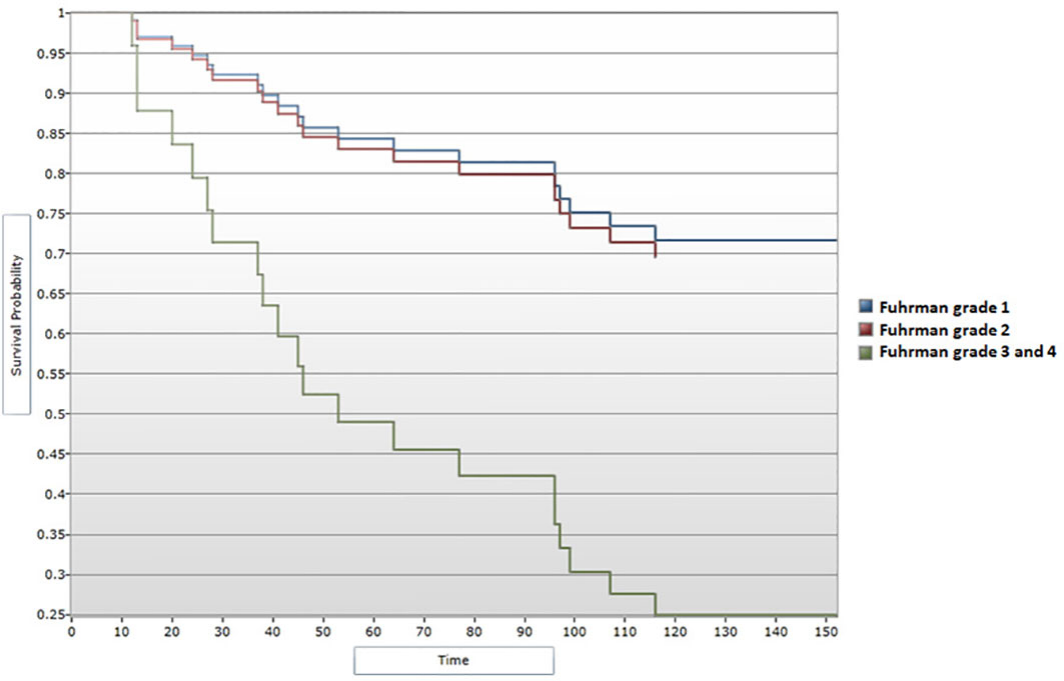
Cox regression analysis between Fuhrman grade and DFS.

**Figure 11:**
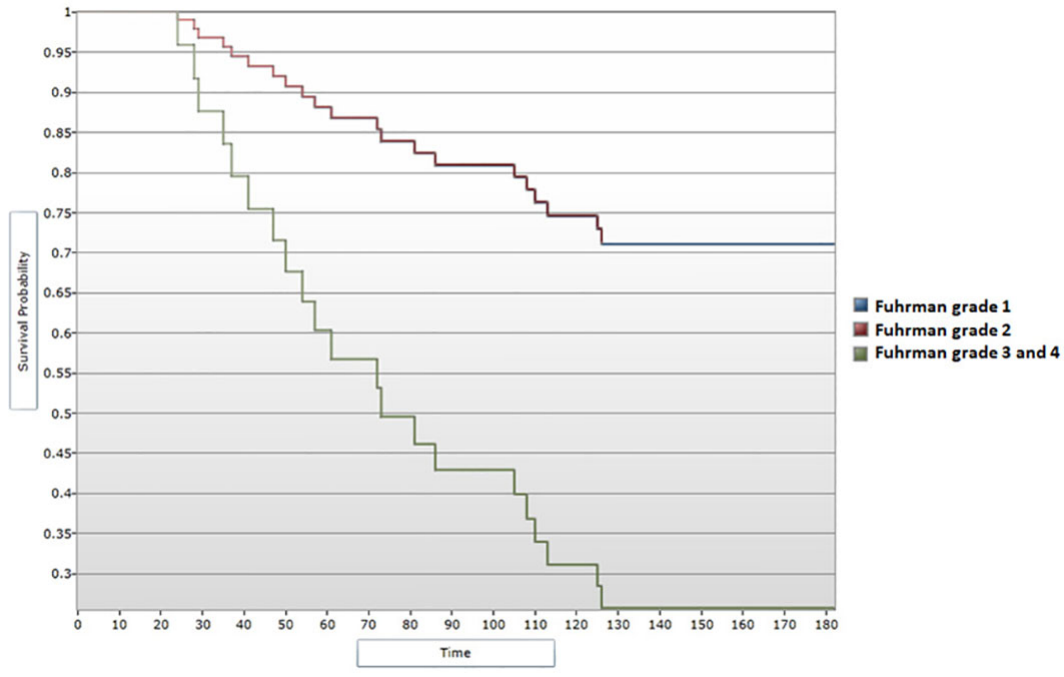
Cox regression analysis between Fuhrman grade and OS.

When assessing survival data in relation to Fuhrman grading for nuclear characterization, median DFS and median OS were shown to follow a descending pattern with increasing of Fuhrman grade. These correlations showed statistical significance (**Figures 8**, **9**). Given the limited number of patients included in the study, during the analysis Fuhrman grade 3 and 4 were taken into consideration simultaneously. Fuhrman grade 1 subgroup presented a median DFS of 130 months and a median OS of 132 months. Patients displaying a Fuhrman grade 2 had a median DFS of 131 months and a median OS of 133 months. Notably, Fuhrman grade 3 + 4 subgroup presented a significantly decreased median DFS and OS, of 45.5 and 66.5 months, respectively. After applying the Cox regression model, the differences remained significant for Fuhrman grade 1 vs. Fuhrman grade 3 in terms of DFS (HR=4,16 95% CI = [1.13, 15.22], p<0.05) (**Figure 10**), as well as OS (HR=3,97, 95% CI = [1.08, 14.54], p<0.05) (**Figure 11**).

Lastly, the presence of diabetes mellitus was assessed as a potential prognostic factor in patients with nmRCC. Based on the data presented by our study, diabetes mellitus comorbidity was not associated with a poorer DFS or OS (p>0.05).

## 4 Discussion

Patients diagnosed with RCC face the particularity of experiencing late relapses, defined by disease recurrence after a disease-free interval of more than 5 years (14). Studies evaluating real-world data on this particular biological behavior of RCC offer valuable insight into potential prognostic factors and clinical outcomes of such cases. The results obtained from real-life studies assist healthcare providers in better managing each case and patient with profiles differing from those portrayed by standardized guidelines. In this context, the current study aims to describe the outcomes of a subgroup of Romanian origin patients who underwent surgical resection with curative intent for nmRCC.

Our study presented a relatively younger population of patients diagnosed with nmRCC, with a median age at diagnosis of 55 years, with literature describing a median age at diagnosis for RCC of 65 years (1). Accounting for more than half of the study population, the percentage of male patients in the current study (70.5%) supports the demographic data confirming that RCC occurs more commonly in male sex individuals (18). In this Romanian subgroup, the majority of patients were diagnosed before the age of 60 years. The reason for the early age in diagnosis in our population could be tied to behavioral and environmental risk factors including tobacco smoking, high body mass index, history of hypertension or exposure to particular carcinogens identified in this geographical region, such as aristolochic acid (19, 20). Large scale studies have attempted to identify risk factors correlating with an increased incidence of cancer rates in younger populations. Among these factors, hereditary predisposition, environmental determinants and notably obesity have been partially recognized as incriminating elements which may lead to higher kidney cancer rates among people up to 40 years of age (21, 22). The spike in kidney cancer diagnosis could also be explained by more frequent incidental diagnosis following high resolution imaging (22). Additionally, certain differences in young-age RCC incidence have also been observed regarding specific ethnic groups (23). Regardless, as cancer diagnosis in younger population leads to important health and socioeconomic consequences, extensive research is warranted in order to identify definitive causes for increasing prevalence of malignancies in young adults (23).

In the current study, the IMDC score was evaluated for patients who experienced recurrence, as well as in patients who remained disease-free for the entire time-period of the study follow-up. The IMDC prognostic model was initially developed to better stratify patients with metastatic RCC eligible for treatment with vascular endothelial growth factor (VEGF) targeted therapies (24). The model was designed in 2002 to include two clinical determinants (a time period of less than 1 year from diagnosis to systemic therapy initiation and a Karnofsky performance status below 80%) and four biological elements (Hb value of less than the lower limit of the normal range, an elevated value of the corrected calcium, and neutrophil and platelet count higher that the upper limit of the normal range) (17). The prognostic model was further validated on large-scale cohorts, leading to an extensive application of the IMDC criteria in clinical trials. The criteria was successfully used to determine survival outcomes correlated to the three prognostic groups identified when applying the score, meaning favorable, intermediate and poor risk (25). Recent studies have also confirmed the utility of the IMDC prognostic model for real-world populations in the current era of immune-oncology therapies and newer anti-angiogenic agents (24). In our study, given the small cohort, patients were attributed one of the following risk groups: favorable for patients evaluated as having a favorable IMDC score, and unfavorable for patients with and IMDC score assessed as intermediate or poor. The predefined favorable risk group was associated with a longer OS as well as DFS, confirming the utility of the IMDC prognostic model in identifying patients at risk of disease recurrence.

NLR has been extensively investigated as a potential prognostic factor in renal oncology. Research has indicated a strong correlation between a high NLR value and poor survival outcomes in both metastatic and non-metastatic cases (26). Real-world studies carried out in diverse populations with smaller-sized cohorts have also repeatedly confirmed the utility of NLR evaluation as a prognostic factor for unfavorable outcomes in RCC (27–29). NLR has also been studied in the context of immunotherapy administration in the setting of RCC, proving that increased values associate with poor outcomes in patients treated with immune checkpoint inhibitors (30). The correlation proved significant with regard to all survival indicatives, however, a clear cut-off value is yet to be accepted. Different studies define contrasting cut-off values for increased NLR corresponding to unfavorable oncologic outcomes, with values ranging from 2.5 to ratios as high as 5 (31–36). The results of the current study align with those described in literature when evaluating the relationship between NLR and DFS, with higher values translating into decreased DFS. However, the study failed to confirm a strong correlation between the investigated ratio and OS, the result potentially being influenced by the small cohort presented in our research. Together with NLR, PLR has also been investigated as a promising prognostic factor in RCC. Vast meta-analysis have concluded its use in determining patients facing an unfavorable outcome, yet research is still warranted in order to better define PLR as biomarker (8, 37). A high PLR value was correlated with poorer survival outcomes in metastatic cases as well as non- metastatic, with cut-off values ranging from 140 to 210 (8, 37–42). A different study, however, has failed to demonstrate the applicability of PLR as a prognostic biomarker in the adjuvant setting (43), and some of the research included in meta-analysis were considered to have various limitations (37), making further research imperative. One research has identified PLR as a stronger predictor of OS compared to NLR (44), while a more recent investigation established that NLR outperforms other biological biomarkers in predicting metastatic RCC outcomes, notably in the setting of immunotherapy administration (45). In the current study, the value of PLR was not found to statistically correlate with DFS and OS, nevertheless, the limited number of patients included in the study requires further consideration. Various biological markers have long been investigated to associate with survival outcomes in RCC. One of the markers intensively studied in the setting of RCC is Hb levels, research showing that lower levels of Hb correlate with shorter survival in patients diagnosed with RCC in the setting of both metastatic and non- metastatic patients (46–48). In addition, research has noted the importance of monitoring dynamic changes in Hb levels during treatment in an attempt to better predict survival outcomes of patients undergoing therapy for RCC (49). However, the current research did not successfully confirm a correlation between Hb levels and survival prognosis.

Tumor characteristics substantially determine the management and outcome of each case. From a staging perspective, tumor dimensions influence survival of patients with metastatic RCC (50), as well as that of patients presenting with localized disease (51–53), with an increase in primary tumor dimensions leading to a worse prognosis. The results of the current study partially support these findings, having proved a correlation between tumor size and DFS, but failing to show a parallel between the dimensions and OS. In addition, Fuhrman nuclear grade was also evaluated. Literature has identified high Fuhrman nuclear grade as a strong biologic marker indicative of an unfavorable prognosis in patients with RCC (54), correlation also supported by the results obtained in the current study.

Diabetes mellitus has been incriminated as an independent risk factor in RCC development (55, 56). Numerous studies have looked into diabetes mellitus as a potential prognostic factor for RCC outcomes. In more recent publications, type 2 diabetes was found to negatively impact recurrence rates, metastatic progression and overall survival in patients diagnosed with RCC (57, 58). However, earlier research did not prove an association between patients with pre-existing diabetes mellitus undergoing surgical intervention for kidney tumor masses and disease-specific outcome in the setting of localized RCC (59, 60). Further investigating this particular correlation on the cohort included in the current study, data did not identify pre-existing type 2 diabetes as a negative prognostic factor for survival outcomes in patients with nmRCC, therefore warranting additional research into the subject overviewing larger populations.

Having recognized the particular biological behavior of RCC exposing patients to the risk of disease recurrence after 5 years from nephrectomy (61, 62), our study analyzed DFS data and looked into correlations between late recurrences and potential predictive determinants. A considerable percent (42.3 %) of the 26 patients experiencing disease recurrence presented late relapses, 60 months after the surgical intervention with curative intent. This result brings valuable insight into the bio characteristics of RCC. According to the research carried out surrounding the topic of late relapses, patients developing cancer recurrence after 5 years since surgical intervention present a better overall prognosis and more favourable disease particularities (14, 63). Additionally, cases of very late relapses have also been described, with disease recurrence presenting after 10 years of surveillance (63, 64). Our study proved that DFS does indeed correlate with certain factors investigated, particularly with the IMDC score, NLR, tumor size and Fuhrman nuclear grade. To further extend our data, the metastatic patterns of RCC following recurrence were investigated. Similar to previous data (65), the most common sites for metastasis identified in our cohort were the lung, bone and lymph nodes. Less frequent metastatic sites were also described in the cases evaluated in our research, finding disease spreading in endocrine organs (adrenal gland, thyroid, pancreas) and other uncommon sites such as pleura, pericardium, soft tissue (65–67).

The current research does face a number of limitations. Given the fact that the current study was designed as a real-world analysis, it is limited by the retrospective nature of the investigation. In addition, the study was conducted non-randomized, on a small number of participants from a single Romanian institution. The limited sample size which makes the object of the current study may weaken the statistical power of the results obtained, as well as restricting these findings from further extrapolation (68). Furthermore, the modest number of patients included may not offer an exhaustive characterization of the general population questioned in the study, presuming the paper underpowered (69). However, certain strengths defining small sample studies may warrant acknowledgement, in particular: the quickness in data collection and assessment, limited costs required for investigating a primary hypothesis, easier completion of institutional and ethical boards requirements. Additionally, smaller studies generally demand fewer centres to be included in one inquiry (70). The current study included one Romanian centre, as The Oncology Institute "Prof. Dr. Ion Chiricuţă" from Cluj‐Napoca represents an institution of reference on a national level. In this context, the patients included in the study could accurately portray baseline characteristics of the general Romanian population which this study attempts to reproduce. Moreover, the study was not designed to include a control group, as the aim of this investigation was to define potential prognostic factors that could cause late disease recurrence in a defined cohort. Lastly, during the collection of study information, the investigators were faced with gaps, rendering this particular study potentially less rigorous. However, despite these limitations, contributing to the already available body of knowledge is crucial for strengthening scientific data and encouraging further research into the field.

## 5 Conclusions

After an in-depth analysis of the results provided during our research, our findings partially support the body of knowledge previously described in literature. The current study associates an unfavorable IMDC score, a high NLR value, an elevated tumor dimension and a high Fuhrman nuclear grade with a shorter DFS following surgical removal of the tumor mass with curative intent. In addition, the study successfully illustrated the particular biological characteristic of RCC, demonstrating the reality of late relapses with a significant percentage of patients experiencing disease recurrence after more than 5 years since undergoing nephrectomy. The conclusions drawn from this study align to a certain degree with those presented in previous research, calling for a quick development of models that could identify patients who would benefit from a longer follow-up timeline. However, given the small sample included for evaluation, the positive results, as well as those not showing scientific significance, warrant further assessment. Additionally, literature provides conflicting results regarding certain prognostic biomarkers, particularly PLR and pre-existing diabetes mellitus in patients diagnosed with RCC. Furthermore, clear cut-off values for biological biomarkers (NLR, PLR, Hb) have yet to be defined in order to better establish prognostic models. In this context, the urgent need for further research into prognostic factors that could predict the behaviour of RCC is apparent. Last but not least, it is important to identify ethnic groups with higher susceptibility to develop RCC from younger ages in the hope of developing tailored screening programs that could reduce the socioeconomic burden of cancer in young populations.

## Data Availability

The original contributions presented in the study are included in the article/supplementary material. Further inquiries can be directed to the corresponding author.
